# Supplementary material for: Mechanoregulated inhibition of formin facilitates contractile actomyosin ring assembly
Source: Nat Commun. 2017 Sep 26;8:703. doi: 10.1038/s41467-017-00445-3 (PMC5614989; doi:10.1038/s41467-017-00445-3)
Supplement: Supplementary file 1 — Supplementary Information [file 41467_2017_445_MOESM1_ESM.pdf]

Description of Supplementary Files

File Name: Supplementary Information
Description: Supplementary Figures, Supplementary Tables and Supplementary References

File Name: Supplementary Movie 1
Description: **Dual-colour TIRFM movie showing *in vitro* reconstituted Search-Capture-Pull from beads containing myosin Myo2 and formin Cdc12.** Yellow and white circles mark the position of the SNAP549-labeled formin Cdc12 bead and Myo2 beads, respectively. Yellow arrowheads indicate the searching actin filament pointed end. The brightest centroids of both Cdc12 and Myo2 beads were followed during Capture-Pull and the resulting traces reflecting effective bead coalescence are shown at the end of the movie. The time interval between frames is 10 s. Display rate: 150X. Movie relates to Fig. 1b–d.

File Name: Supplementary Movie 2
Description: **Second example of a dual-colour TIRFM movie showing *in vitro* reconstituted Search-Capture-Pull from beads containing myosin Myo2 and formin Cdc12.** Yellow and white circles mark the position of the SNAP549-labeled formin Cdc12 bead and Myo2 beads, respectively. Yellow arrowheads indicate the searching actin filament pointed end. The brightest centroids of both Cdc12 and Myo2 beads were followed during Capture-Pull. The time interval between frames is 5 s. Display rate: 75X. Movie relates to Supplementary Fig. 1a–c.

File Name: Supplementary Movie 3
Description: **Dual-colour TIRFM movie showing that active pulling by myosin Myo2 is required to inhibit formin Cdc12.** SNAP549-labeled Cdc12 adhered to the coverslip surface is marked with a yellow circle. The NEM-Myo2 bead is marked with a white circle. Yellow arrowheads indicate the searching actin filament pointed end. The time interval between frames is 5 s. Display rate: 75X. Movie relates to Fig. 2e,f.

File Name: Supplementary Movie 4
Description: **Dual-colour TRIFM movie showing that even under complete tension formin mDia2 remains fully active during myosin Myo2-mediated Capture-Pull.** SNAP549-labeled formin mDia2 beads are marked with yellow circles. Myo2 beads are marked with white circles. Yellow arrowheads indicate the searching actin filament pointed end. The time interval between frames is 5 s. Display rate: 50X. Movie relates to Fig. 3a,b.

File Name: Supplementary Movie 5
Description: **Simulations of Search-Capture-Pull at *in vivo* conditions.** Formin node (red) polymerizes two actin filaments (white) that are captured by a myosin node (cyan). Actin is polymerized at *in vivo* rates (200 nm s-1) and myosin was set to walk at its saturated rate (540 nm s-1, Supplementary Fig. 2a), with physiological drag forces. Movies stop once nodes have coalesced to a distance of 50 nm. *Left*, formin-mediated polymerization is inhibited, whereas on the *right* formin-mediated polymerization is not inhibited during myosin Capture-Pull. The time interval is 0.1 s. Display rate: 1.5X. Movies relate to Supplementary Fig. 4 and Fig. 7i.

File Name: Supplementary Movie 6
Description: **Live-cell time-lapse series of multiple cells showing that mechanoinsensitive mutant *mDia2* fission yeast cells exhibit ring assembly defects.** *Left*, control *cdc12* cells assemble rings from canonical precursor cytokinesis nodes, while for mutant *mDia2* cells (*right*) nodes collapse into clumps. As node marker, cells express myosin light chain Rlc1 (-3GFP). Z-stacks of four slices were

acquired every 15 seconds. Time zero is the start of imaging. Display rate: 149X. Scale bar, 5 µm. Movie relates to Fig. 7a.

File Name: Supplementary Movie 7
Description: **Live-cell time-lapse series of mutant *mDia2* fission yeast cells showing that nodes aggregate into clumps.** *Upper panel,* control *cdc12* cells form cytokinesis nodes that coalesce into a sharp ring, while in mutant *mDia2* cells (*lower panel*) nodes (red open arrowheads) rapidly aggregate into clumps (blue filled arrowheads). As node marker, cells express myosin light chain Rlc1 (-3GFP). Z-stacks of four slices were acquired every 15 seconds. Display rate: 75X. Scale bar, 5 µm. Movie relates to Fig. 7a–c.

File Name: Peer Review File

Supplementary Figures

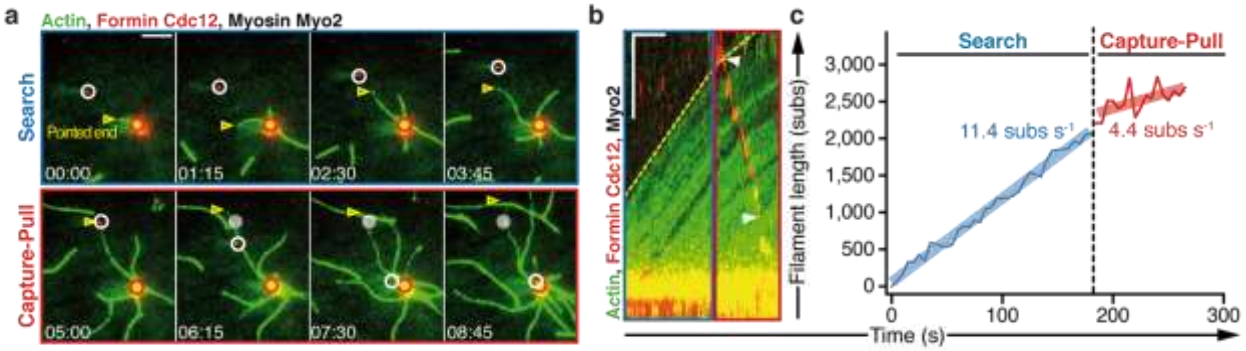

Supplementary Figure 1. Inhibition of formin Cdc12-mediated F-actin elongation during *in vitro* reconstituted Myo2 Capture-Pull. (**a–c**) Dual-colour TIRFM micrograph time-series (min:s) (a, scale bar=5 µm), Kymograph (b, scale bars=100 s and 5 µm in x- and y-direction, respectively) and filament elongation rates (c), of an actin filament elongating (searching, blue) from a SNAP-549 labelled Cdc12-associated bead (red circle) that is captured and pulled (red) by a Myo2-associated bead (white circle). For time series, initial bead positions are marked with opaque filled circles. Blue and red outlines in (a and b) delineate Cdc12-mediated actin filament elongation before (Search) and during Capture-Pull. Dashed yellow lines mark actin filament elongation rates, and white arrowheads mark the Myo2 bead positions at onset and end of Capture-Pull (Supplementary Movie 2). Shaded lines (c) represent the regression line fits for the respective filament elongation rate trace during the Search and Capture-Pull phase. Average formin elongation rates are listed in Supplementary Table 1.

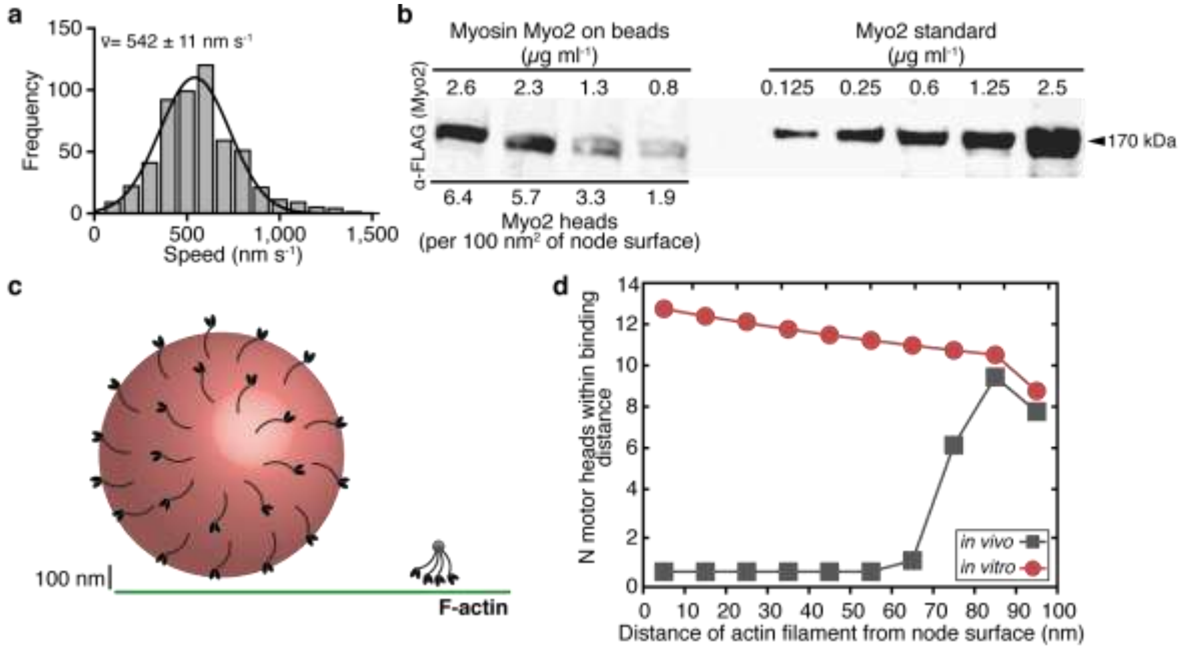

Supplementary Figure 2. Quantitative analysis of myosin Myo2 velocities and head density on biomimetic nodes. (a) Myo2 F-actin gliding speeds. Myo2 gliding filament assay data were plotted in a histogram and fitted to a single Gaussian, yielding an average saturated Myo2 speed of 0.54 ± 0.01 µm s^{-1} (Mean ± S.E.M., n=10 filaments). (**b**) Quantitative immunoblotting of Myo2 head number density on coated beads. Myo2 was detected using anti-FLAG primary antibody on a dilution series of Myo2-coated beads (4 left lanes) and a Myo2 standard of known concentrations (5 right lanes). (**c**) Cartoon of biomimetic a Myo2-coated bead (1 µm diameter, red) and an *in vivo* node (50 nm diameter, black) encountering a green actin filament. For the calculation of motor head density and number of engaged motor heads, we assumed the *in vivo* node to be of spherical shape. Importantly, only half of the *in vivo* node surface area was assumed to be occupied by myosins (black portion) with the other half masked (grey portion) due to association with the plasma membrane. The biomimetic bead and *in vivo* node are drawn to scale. (**d**) The number of myosin heads (y-axis) that can be engaged with an actin filament at a given time was plotted for a range of filament-to-node surface distances (x-axis), taking the geometric constraints of an object of spherical shape into consideration (see Methods). With a Myo2 stalk length of ~90 nm long[1,2], we estimate that 7 to 9 and 9 to 12 Myo2 heads are available for engaging an actin filament for *in vivo* nodes and *in vitro* beads, respectively.

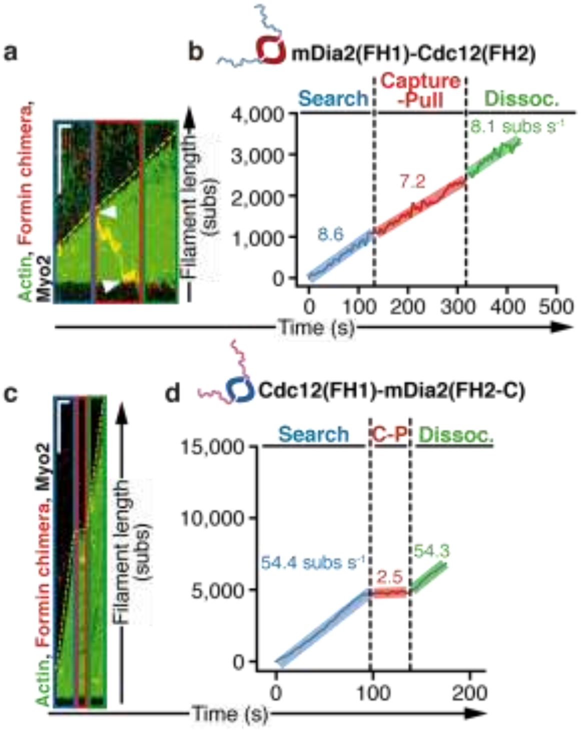

Supplementary Figure 3. Formin Cdc12 mechanosensitivity maps to the FH1 domain. (**a** and **c**) Kymographs of Search-Capture-Pull events for actin filaments elongated by the chimeric formins mDia2(FH1)-Cdc12(FH2) and Cdc12(FH1)-mDia2(FH2-C). Blue, red and green outlines in (a and c) delineate Cdc12-mediated actin filament elongation before (Search), during and after Capture-Pull, respectively. Dotted yellow lines mark filament elongation rates, and white arrowheads mark the Myo2 bead position at the beginning and end of Capture-Pull. Scale bars=50 s and 5 µm in x- and y-direction, respectively. (**b** and **d**) Formin-mediated filament elongation rates over time for the filaments shown in (a and c) before (blue), during (red) and after (green) Capture-Pull. Shaded lines represent the regression line fits for the respective filament elongation rate trace during the Search, Capture-Pull and Dissociation phase. Average formin elongation rates are listed in Supplementary Table 1.

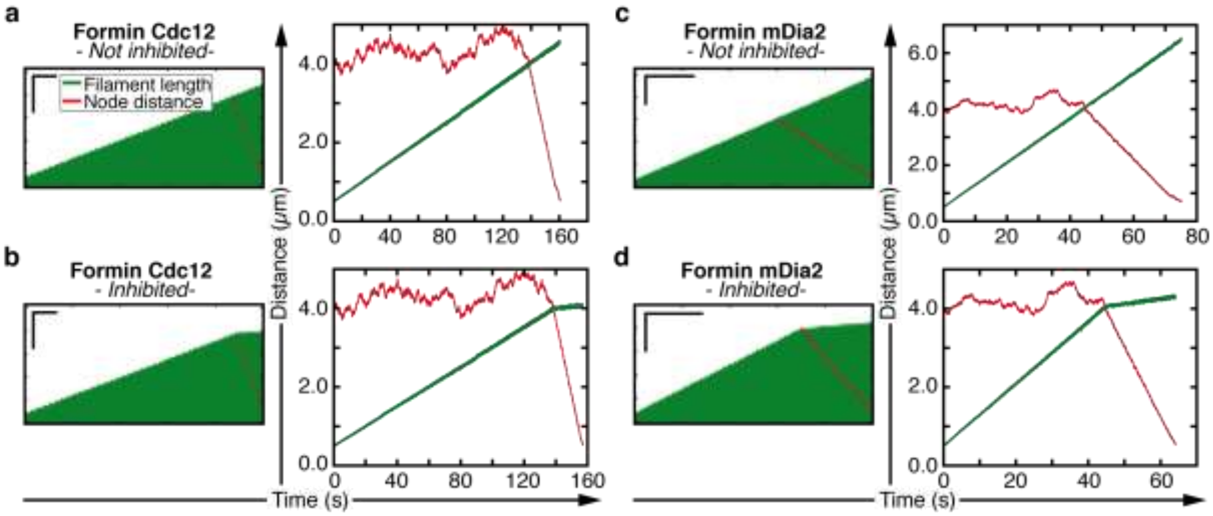

Supplementary Figure 4. Mathematical modelling of Search-Capture-Pull under *in vitro* reconstitution conditions. We adapted a model of Search-Capture-Pull (see Methods section) to match the conditions of the *in vitro* experiment, including larger biomimetic nodes (beads), lower viscosity, and placement of myosin and formins on separate beads. Formin beads start with two filaments of length 0.5 µm and polymerize actin at a rate of 25 nm s^{-1} (~Cdc12 rates) and 80 nm s^{-1} (~mDia2 rates). Beads were initially separated by 5 µm. The viscosity acting on the beads is 0.22 Pa s. (**a–d**) Kymographs and plots, along with the accompanying Supplementary Movie 5, reveal that the Capture-Pull behaviour observed in experiments (Fig. 1 and 2) are recapitulated *in silico*. Scale bars=15 s and 1.5 µm in x- and y-direction, respectively. (**a** and **c**) Examples of dynamics where after Capture the formin is not inhibited. (**b** and **d**) Examples where the formin on the bead is inhibited by pulling force upon Capture. All four simulations have the same random seed (i.e. the sequence of pseudo-random numbers used to simulate the dynamics are identical for (a–d). Hence, the data are identical before Capture when comparing (a) with (b) and (c) with (d).

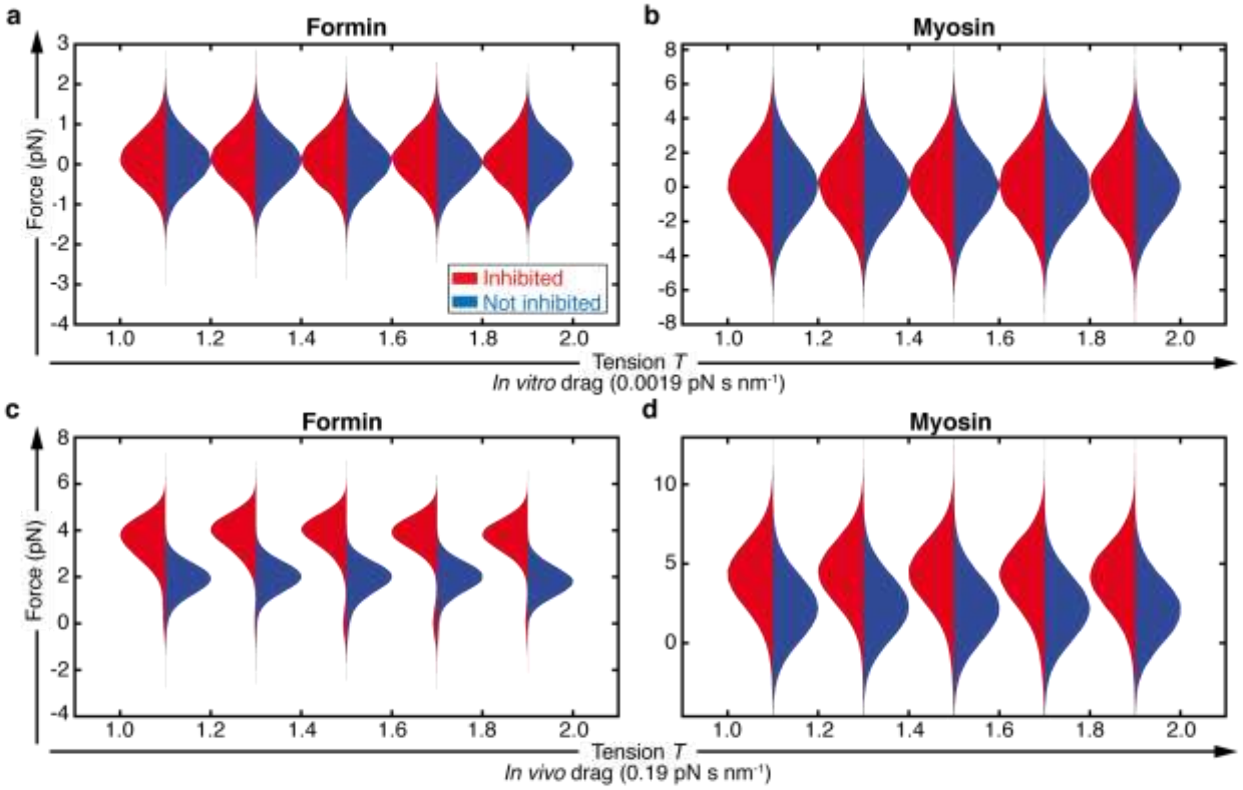

Supplementary Figure 5. Numerical simulation of applied pulling forces under *in vitro*- and *in vivo*-like conditions. Based on the model described in the Methods section, simulations were run with biomimetic nodes (beads) separated by 5 µm, using drag (i.e. friction) levels that recapitulate the experimental *in vitro* drag with an effective viscosity of 0.22 Pa s (**a** and **b**), or the 100-times higher *in vivo* drag (**c** and **d**). Filament tension (*T*) is defined as the ratio of the actin filament length to the distance between the nodes, where higher values reflect more apparent filament slack (see Fig. 6a). The magnitude of force (y-axis) that is felt by the formin (**a** and **c**) and myosin (**b** and **d**) are graphed as histograms in the dependence of *T* (x-axis) under conditions where formin-mediated actin filament assembly is inhibited (red) or not inhibited (blue). With decreasing filament tension (increasing *T*), the mean force decays slowly and at the same time is transmitted across the entire filament length, which is consistent with the experimental findings presented in Supplementary Fig. 6a,b.

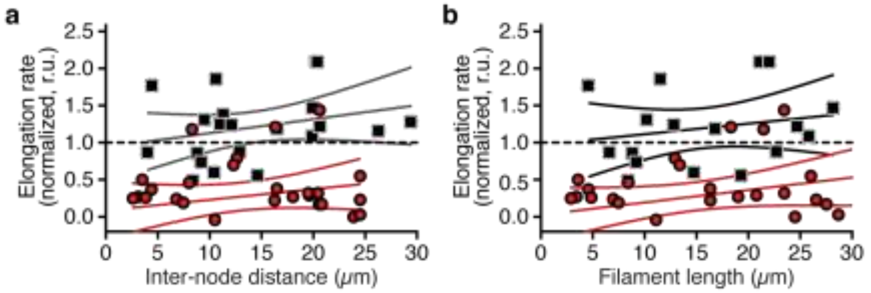

Supplementary Figure 6. Cdc12 is inhibited even over a large range of Capture-Pull distances while formin mDia2 activity is largely unaffected. (**a** and **b**) Comparison of formin Cdc12 (red filled circles)- and mDia2 (black filled squares)-mediated filament elongation rates (normalised) as a function of increasing filament contour length (a) and node-to-node distances (b) between formin- and myosin-beads (n_{Cdc12}=19 independent events, n_{mDia2}=15 independent events). Red or dark grey regression lines, as well as confidence intervals (α=0.05), are included for Capture-Pull events with formins Cdc12 or mDia2.

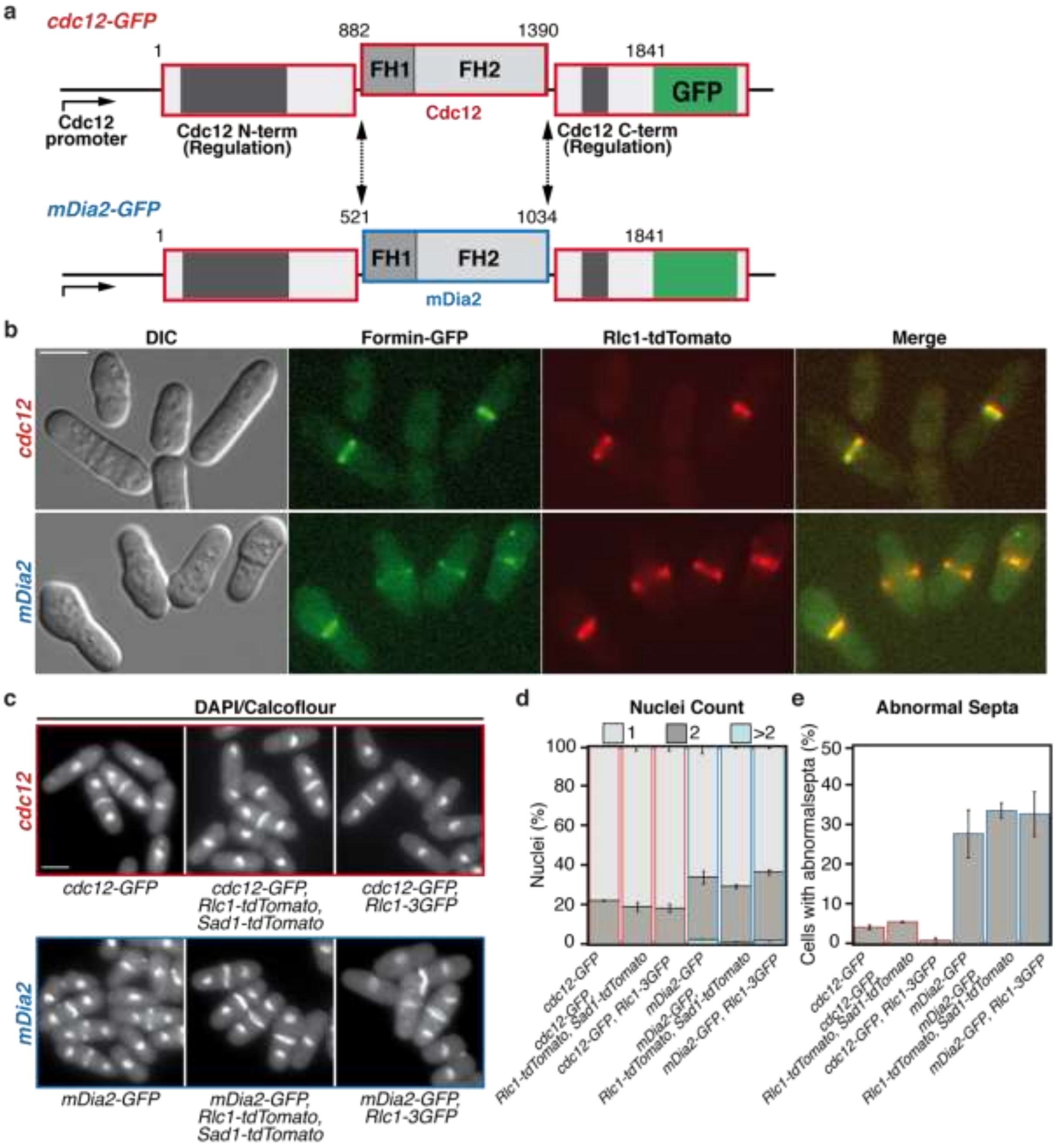

Supplementary Figure 7. Characterization of fission yeast cells expressing a mechano-insensitive cytokinesis formin. (**a**) Schematic showing the engineering of a fission yeast strain where the actin assembly FH1FH2 domains of Cdc12 were replaced with the FH1FH2 domains of the mechanoinsensitive (uninhibited) mDia2 (Cdc12N-mDia2FH1FH2-Cdc12C). The N- and C-terminal regulatory regions of wild-type *cdc12* are unchanged. (**b**) DIC and fluorescent micrographs of control *cdc12* and mutant *mDia2* (formin-GFP, Rlc1-tdTomato) cells during cell division. Scale bar=5 µm. (**c**) Micrographs of control *cdc12* and mutant *mDia2* cells fixed and stained with DAPI (nuclei) and Calcofluor (septa). Scale bar=5 µm. (**d** and **e**) Quantification of general cytokinesis defects. Control or mutant strains with different fluorescent markers exhibit

similar defects. Error bars indicate S.E.M. of two replicates of n $\geq$300 cells. Percentage of cells with the indicated number of nuclei (d) and with abnormal septa (e).

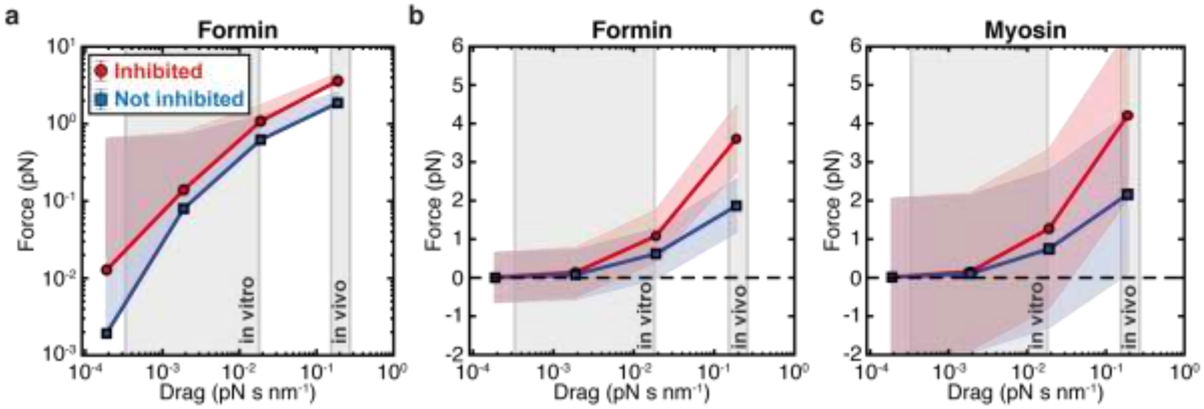

Supplementary Figure 8. Pulling forces applied on formin-bound filaments under *in vitro*- and *in vivo*-like conditions. The forces on the formin-bead (**a**, log scale and **b**, linear scale) generated by the myosin (**c**) are computed from simulations where actin is polymerized at 80 nm s^{-1} and the unloaded myosin speed is 200 nm s^{-1}, with increasing drag applied to the nodes and other parameters held constant. For each simulation, the average and standard deviation of force on the formin node was computed. These values were then averaged over all sets of simulations performed, and shown as points and shaded error bars, respectively. As shown in Supplementary Fig. 5, the inhibition of the formin (red circles) increases the force felt by formin and the force generated by the myosin. Under *in vitro*-like conditions, due to the overdamped low Reynolds number behaviour, we observe that most forces experienced by the formin are sub-piconewton. However, under *in vivo* conditions, much larger forces should be expected on the formin. We note that under *in vivo* drag, the myosin exerts approximately 4 pN of force when pulling on a filament that is not elongating. Thus, while we used a different motor model than in previous work[3] we recapitulate the same forces under the same conditions.

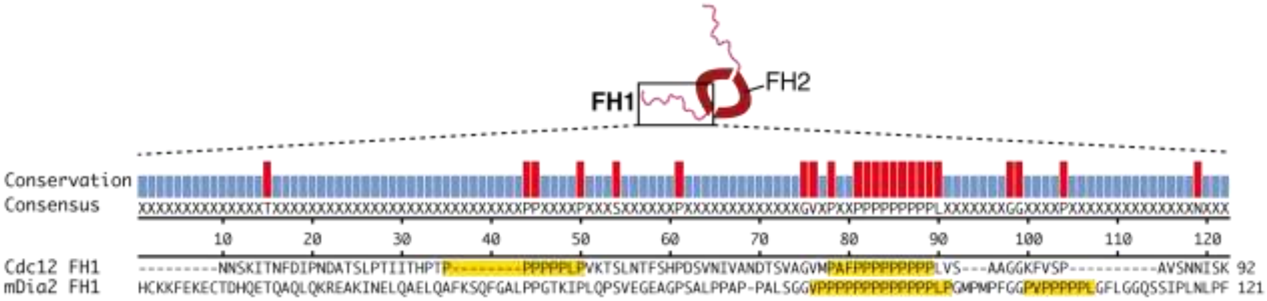

Supplementary Figure 9. Sequence comparison for formin homology 1 (FH1) domains of formins Cdc12 and mDia2. The sequences of formin homology 1 (FH1) domains from *S.pombe* formin Cdc12 and *M.musculus* formin mDia2 were aligned in ClustalW mode, using the blosum62 amino acid table. A profilin-binding proline-rich track (yellow shading) was defined as more than three consecutive proline residues[4,5] and proline-rich tracks with more than 12 consecutive prolines were counted as multiple tracks. Consensus (red) and non-consensus (blue) residues are indicated in the top row. While the two FH1 domains both contain two proline-rich tracks and are similar in length (92 vs. 121 amino acid residues for Cdc12 vs. mDia2), there is no overall sequence similarity between the two. The main differences between the two are that Cdc12 FH1 is 25% shorter and its two profilin-binding proline-rich tracks (N-terminal PBT1 and C-terminal PBT2) are considerably farther away from the FH2 domain than it is the case for mDia2 (20% for PBT1 and 60% for PBT2 vs. 14% for PBT1 and 26% for PBT2 relative to the total FH1 domain length of Cdc12 vs. mDia2).

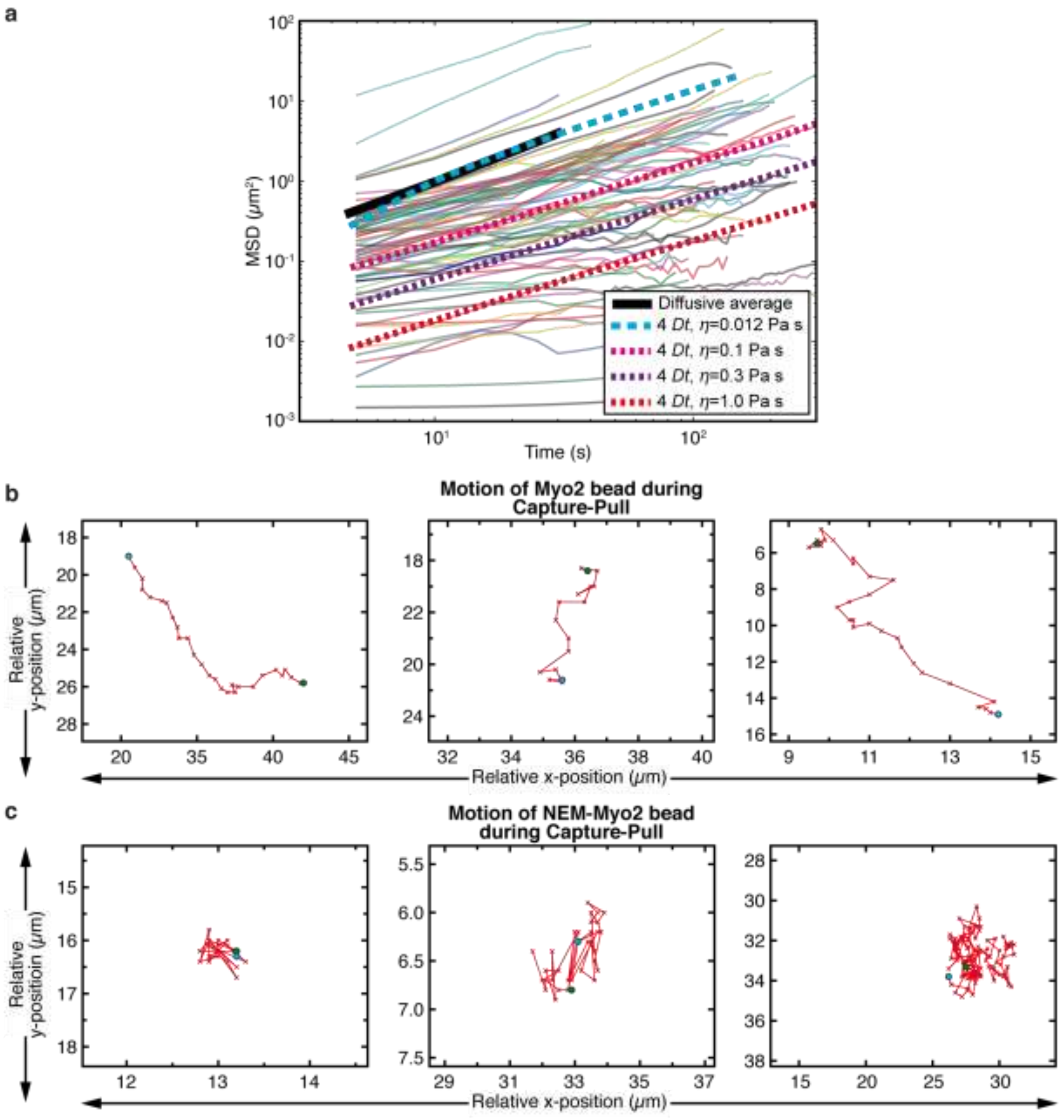

Supplementary Figure 10. Diffusive behaviour of Myo2 beads before and during Capture-Pull. (**a**) Mean-square-displacement (MSD) of myosin-coated beads prior to capture for n=76 events spanning all of the experiments described in this paper. Fits of log MSD vs. α*log t up to t=30 allowed us to determine via the parameter α whether the bead was diffusing ($\alpha \sim 1$**)** or sub-diffusing (we chose α <0.8 as a cut-off). The black solid line shows the average of data with α> 0.8. Fitting this line to MSD(t) vs. 4Dt resulted in an estimated viscosity of 0.012 Pa s for the medium (see Methods for further details). Additional dashed lines are a guide to the eye showing diffusive behaviour (MSD=4Dt) for higher viscosities. (**b** and **c**) Position of myosin-coated beads *during capture* for three typical experiments where the myosin is active (**b**) or

inactivated by NEM (**c**). The bead starts at the position marked by a green circle and ends at the position marked by a blue circle, with each cross-mark representing the position of the bead in the subsequent frame (interval 5 seconds). There is a clear qualitative difference, as the active beads translate over large distances in shorter times than the inactivated beads. Further, it is obvious that inactivated myosin beads do not translocate away from the formin bead with any substantial rate due to the force of actin polymerization. Rather, the polymerization force buckles the extended filament (e.g. in Supplementary Movie 3). Therefore, it seems unlikely that the force on the formin pushing against an inactive myosin bead is anywhere close to the force that is felt by the formin under active myosin pulling conditions.

Supplementary Tables

Supplementary Table 1. Formin-mediated actin assembly rates during Search-Capture-Pull

Formin (n=independent experiments)	Elongation rates (subs s^{-1}, Median ± S.E.M.)		
	Search	Capture-Pull	Dissociation
**SNAP-Cdc12(FH1FH2) †, n=11**	9.6 ± 1.12	2.8 ± 0.93	10.25 ± 1.49
**SNAP-Cdc12(FH1FH2) *†, n=11**	18.5 ± 2.24	15.1 ± 1.83	15.5 ± 2.69
**SNAP-mDia2(FH1FH2) †, n=9**	28.2 ± 3.82	34.4 ± 3.55	28.75 ± 7.92
**SNAP-mDia2(FH1FH2) #†, n=6**	7.95 ± 1.1	8.5 ± 1.51	20.1 ± 6.02
**SNAP-Cdc12(FH1)-mDia2(FH2) ‡, n=10**	36.5 ± 4.65	4.55 ± 2.12	46.1 ± 8.67
**SNAP-mDia2(FH1)-Cdc12(FH2) ‡, n=11**	11.4 ± 2.24	9.45 ± 2.02	8.9 ± 2.18
**SNAP-Cdc12(FH1FH2) ‡, n=7**	20.5 ± 3.5	5.6 ± 1.79	19.3 ± 4.56
**Cdc12(FH1FH2)-SNAP §, n=11**	14.6 ± 4.76	13.55 ± 2.24	23.3 ± 3.2
**mDia2(FH1FH2)-SNAP §, n=8**	29.25 ± 3.1	36.3 ± 2.74	29.2 ± 6.73

* in the presence of NEM-Myo2 (only Capture, no pulling)

# at decreased 1 μM G-actin concentrations

† Formin attached to beads (via FH1 domain)
‡ Formin attached to glass surface (via FH1 domain)
§ Formin attached to glass surface (via FH2 domain)

Supplementary Table 2. Velocities at which beads coalesce during Capture-Pull

Condition	Net velocities for bead pulling (nm s^{-1}, Mean ± S.E.M.)
**SNAP-Cdc12(FH1FH2) †**	93 ± 17 (n=11)
**SNAP-mDia2(FH1FH2) †**	155 ± 25 (n=6)
**SNAP- mDia2(FH1FH2 #†**	87 ± 18 (n=5)
**SNAP-Cdc12(FH1)-mDia2(FH2) ‡**	125 ± 25 (n=4)
**SNAP-mDia2(FH1)-Cdc12(FH2) ‡**	91 ± 8 (n=7)
**SNAP-Cdc12(FH1FH2) ‡**	69 ± 11 (n=19)
**Cdc12(FH1FH2)-SNAP §**	72 ± 12 (n=21)
**mDia2(FH1FH2)-SNAP §**	143 ± 27 (n=17)

# at decreased 1 μM G-actin concentrations

† Formin attached to beads (via FH1 domain)
‡ Formin attached to glass surface (via FH1 domain)
§ Formin attached to glass surface (via FH2 domain)

Supplementary Table 3. Constants used in the mathematical modelling

Constant	Value	Comment
**Time step (*dt*)**	5×10^{-6} s	
**Event time (t_{event})**	0.001 s	
**Initial bead-bead distance**	5,000 nm / 600 nm	*in vitro / in vivo*
**Actin radius (*a*)**	3.5 nm	
**Actin segment length (l_0)**	250 nm / 50 nm	*in vitro / in vivo*
**Actin bending coefficient (κ)**	82.28 pN/rad^2	
**Actin stretch coefficient (k_{actin})**	0.2 pN nm^{-1}	see [6]
**Viscosity on actin (η_{actin})**	0.012 Pa s	
**Viscosity on beads (η_{beads})**	0.22 Pa s / 215 Pa s	*in vitro / in vivo*
**Rotational constant (k_{rot}/l_0)**	10 pN/rad	see [6]
**Bead diameter (*d*)**	1,000 nm / 25 nm	*in vitro / in vivo*
**Polymerization speeds (v_{pol})**	25 and 80 nm s^{-1} / 200 nm s^{-1}	*in vitro / in vivo*
**Formin force threshold (F_{cut})**	1×10^{-4} pN / 10 pN	see [6]
**Myosin speed (v_{myo})**	150 – 400 nm s^{-1}	
**Myosin force constant (k_{myo})**	1.0 pN nm^{-1}	
**Myosin force threshold (F_{thresh})**	5.0 pN	
**Myosin capture radius ($R_{capture}$)**	550 nm / 55 nm	*in vitro / in vivo*

Supplementary Table 4. Fission yeast strains used in this study

Strain name	Genotype	Reference
**KV496**	*h?, cdc12Δ::KanR, cdc12(Nterm)-cdc12(FH1FH2)-cdc12(Cterm)-GFP::ura4+, rlc1-tdTomato-natMX6*	This study
**KV506**	*h?, cdc12Δ::KanR, cdc12(Nterm)-mDia2(FH1FH2)-cdc12(Cterm)-GFP::ura4+, rlc1-tdTomato-natMX6*	This study
**KV956**	*h?, cdc12-mGFP, rlc1-3GFP-kanMX6, ade6-M210, leu1-32, ura4-D18*	This study
**KV957**	*h?, cdc12Δ::KanR, cdc12(Nterm)-mDia2(FH1FH2)-cdc12(Cterm)-GFP::ura4+ (chimera integrated at ura locus), rlc1-3GFP-kanMX6, ade6-M210, leu1-32*	This study
**KV940**	*h?, cdc12-mGFP, rlc1-tdTomato-natMX6, sad1-tdTomato-natMX6, ade6-M210, leu1-32, ura4-D18*	This study
**KV962**	*h?, cdc12Δ::KanR, cdc12(Nterm)-mDia2(FH1FH2)-cdc12(Cterm)-GFP::ura4+ (chimera integrated at ura locus), rlc1-tdTomato-natMX6, sad1-tdTomato-natMX6, ade6-M210, leu1-32, ura4-D18*	This study

Supplementary Table 5. Numerical statistics on bead friction, viscosity and force values

Dimension	Median	1st to 3rd Quartile range
**Friction ζ**	0.002 pN s nm^{-1} (n=53)	0.0003 to 0.011 pN s nm^{-1}
**Viscosity η**	0.19 Pa s (n=53)	0.03 to 1.12 Pa s
**Force $\boldsymbol{F}$**	0.1 pN (n=83)	0.02 to 1.2 pN

Supplementary References

1 Bezanilla, M. & Pollard, T. D. Myosin-II tails confer unique functions in Schizosaccharomyces pombe: characterization of a novel myosin-II tail. *Mol Biol Cell* **11**, 79-91 (2000).

2 Laplante, C., Huang, F., Tebbs, I. R., Bewersdorf, J. & Pollard, T. D. Molecular organization of cytokinesis nodes and contractile rings by super-resolution fluorescence microscopy of live fission yeast. *Proc Natl Acad Sci U S A* **113**, E5876-E5885, doi:10.1073/pnas.1608252113 (2016).

3 Vavylonis, D., Wu, J. Q., Hao, S., O'Shaughnessy, B. & Pollard, T. D. Assembly mechanism of the contractile ring for cytokinesis by fission yeast. *Science* **319**, 97-100, doi:10.1126/science.1151086 (2008).

4 Zeile, W. L., Purich, D. L. & Southwick, F. S. Recognition of two classes of oligoproline sequences in profilin-mediated acceleration of actin-based Shigella motility. *J Cell Biol* **133**, 49-59 (1996).

5 Courtemanche, N. & Pollard, T. D. Determinants of Formin Homology 1 (FH1) domain function in actin filament elongation by formins. *J Biol Chem* **287**, 7812-7820, doi:10.1074/jbc.M111.322958 (2012).

6 Laporte, D., Ojkic, N., Vavylonis, D. & Wu, J. Q. alpha-Actinin and fimbrin cooperate with myosin II to organize actomyosin bundles during contractile-ring assembly. *Mol Biol Cell* **23**, 3094-3110, doi:10.1091/mbc.E12-02-0123 (2012).
